# Effect of temperature on the life cycle of *Harmonia axyridis* (Pallas), and its predation rate on the *Spodoptera litura* (Fabricius) eggs

**DOI:** 10.1038/s41598-022-18166-z

**Published:** 2022-09-12

**Authors:** Yasir Islam, Ali Güncan, Xingmiao Zhou, Afifa Naeem, Farhan Mahmood Shah

**Affiliations:** 1 Hubei Insect Resources Utilization and Sustainable Pest Management Key Laboratory, College of Plant Science and Technology, Huazhong Agricultural University, Wuhan, 430070 China; 2 Department of Plant Protection, Faculty of Agriculture, Ordu University, 52200 Ordu, Turkey; 3 Entomological Research Institute, Ayub Agricultural Research Institute, Faisalabad, Punjab Pakistan; 4 Department of Entomology, Faculty of Agricultural Sciences and Technology, Bahauddin Zakariya University, Multan, 60000 Pakistan; 5 National Center for Natural Products Research, The University of Mississippi, University, MS USA

**Keywords:** Developmental biology, Ecology, Physiology, Ecology, Environmental sciences

## Abstract

Biological control is one of the strategies of pest control which is determined by the biological fitness and metabolic rates of the predator species used. Temperature and resource are important factors which influence the role of insects as biocontrol agents. *Harmonia axyridis* is a cosmopolitan and non-specific polyphagous predator. It can survive ecologically diverse environments and exploit multiple preys. This study investigated the effects of temperature on the population parameters of *H. axyridis* and its predation on the eggs of prey *Spodoptera litura*. For this purpose, an age–stage, two-sex life table of the predator was constructed at four constant temperatures, i.e. 15, 20, 25 and 30 °C, under laboratory settings of: 70 ± 5% RH, and 16:8 h (L: D) photoperiod. A computer simulation was then used to project the population and predation responses with respect to temperatures tested. We found that the development of larvae and adult (male/female) stages of *H. axyridis* decreased with colder temperatures (i.e., 15 and 20 °C) but increased with warmer temperatures (25 and 30 °C). The intrinsic rate of increase (*r*) and mean generation time (*T*) were 0.0662 d^−1^ and 79.84 d at 15 °C, 0.0843 d^−1^ and 64.90 d at 20 °C, 0.1067 d^−1^ and 48.89 d at 25 °C, and 0.1378 d^−1^ and 35.55 d at 30 °C, respectively. The mean duration of the total pre-adult stage was 44.26, 32.91, 20.63, and 15.39 d at 15, 20, 25, and 30 °C, respectively. At 30 °C. the finite rate of increase (1.1477 d^−1^) was the highest and the mean generation time (35.55 d) was the shortest. The net predation rate (*C*_0_) was 7935.54, 10,466.28, 10,139.38, and 7126.36 eggs at 15, 20, 25, and 30 °C, respectively. Population and predation projections were proportional to temperature. These findings are important for modelling the population responses of *H. axyridis* to climate change and tailoring integrated pest management strategies to altered climates.

## Introduction

*Spodoptera litura* (Fabricius) (Lepidoptera: Noctuidae) is a cosmopolitan and obnoxious pest of more than 100 crops, ornamentals, and vegetable species^1^. A single mother can lay over 2,000 eggs and huge populations are present when conditions are favourable^2^. Larvae are gregarious leaf eaters, and uncontrolled feeding can induce 100 percent losses in some sensitive crops. Pesticides are primary tools that are used to control this pest, but inappropriate use has several associated problems. It has led to the development of pesticide resistance against routinely used organophosphates, carbamates, pyrethroids, indoxacarb, abamectin, emamectin benzoate, and chlorantraniliprole^3^. As a result of resistance development, it becomes difficult to control the pest and outbreaks occur due to control failures^4.^ Further challenges include potential risks to biodiversity, humans, and the environment. Environmental-friendly strategies such as biological control as replacements of conventionally used pesticides are thus need of time^5–7^.

Biological control is one of the finest means of suppressing pest problems with reduced reliance on synthetic insecticides^8^. Many factors that significantly determine biocontrol fate include species identity, resource quality/quantity, and other ecological considerations^9^. Temperature is primarily important for biological systems through its close link with species metabolic rates and fitness parameters i.e., survival, development, reproduction, handling time, and searching efficiency of predators^10^. Since insects are ectotherms, they rely greatly on temperature for their growth, reproduction, maturation and trophic relations^11^. Insects vary greatly in thermal sensitivity levels, hunting behaviour, and resource-specificity. Assessment of the effect of temperature at individual species level^12^ is typically desired to improve our understanding about temperature regulation of trophic cascades and population dynamics^13^. This will pave a way towards the development of effective biocontrol strategies under altered climates^11^. The use of age-stage, two-sex life table approach, developed by Chi and Liu (1985) and Chi (1988), represents an effective way of studying fitness of the individuals under influence of stressors^14^.

Polyphagous predators can be mass-reared and produced with artificial diets, and alternative resource can increase their numbers in the field^15^. The family Coccinellidae includes a globally widespread group of predatory beetles that are polyphagous^16^ and indicate preference for resource including transitions across kingdoms (Animalia, Fungi, and Plantae) and trophic levels (carnivorous and herbivorous)^17^. Many coccinellids are important predators of hemipterans, including aphids^10,18^, whitefly^19^, mealybugs^20^, scale insects^21^ and immature lepidopterans^22,23^. Resource quality, thermal surroundings, mobility^24^, nutritional status^25^, as well as density and size of the prey^26^ are critical determinants of predator development, biology^16^ and predation parameters^12,27–29^. The biological control effectiveness of the predator can thus be evaluated by combining development, survival rates, and reproductive potential with age-specific predation rates^30^.

The multicolored Asian ladybird beetle, *Harmonia axyridis* (Pallas) (Coleoptera: Coccinellidae), native to Asia, is a globally widespread predator that feeds on many resources, including 77 insect taxa on 85 plant species in 35 families^15,31^. This predator is highly voracious, multi-voltine, greatly dispersed^32^ and has ability to survive complex tropical and sub-tropical environments around the world^31^. *H. axyridis* has been commercially reared and extensively deployed as a potential biocontrol agent in many biocontrol programs worldwide. Its feeding is reported on hemipterans and lepidopterans, including immature stages of *Danaus plexippus* (Lepidoptera: Nymphalidae)^22^, *Anagasta kuehniella* (Lepidoptera: Pyralidae)^33^, *Sitotroga cerealella* (Lepidoptera: Gelechiidae)^34^, *Spodoptera litura*^35^ and *Spodoptera frugiperda* (Lepidoptera: Noctuidae)^36^.

Life table of this predator has been developed on various insects and temperatures^10,33,34^. The population parameters and feeding rate of this predator have been shown to depend on the temperature and resource^30^. Using laboratory experiments, we showed that foraging by *H. axyridis* on the eggs and larvae of *S. litura* (eggs and larvae) is temperature-derived^23,35^. Predator (larvae and adults) increases its foraging with warmer temperatures and decreases with colder temperatures. What remains unexplored is the impact of temperature-influenced feeding on subsequent predator development and predation rate. The present study answers this question by feeding and rearing *H. axyridis* on the eggs of *S. litura* at four constant temperatures by hypothesizing that predation and life history parameters of *H. axyridis* on *S. litura* eggs will be different across four temperatures tested.

## Materials and methods

### Mass rearing of insects

*Harmonia axyridis* population culture was established from a laboratory reared stock at Key Laboratory of Huazhong Agricultural University (HZAU), Wuhan, China, during February 2019. The adult insects were grouped in transparent plastic boxes (12 cm × 16 cm × 7 cm) with moistened cotton swabs, and reared on ad libitum diet of *Acyrthosiphon pisum* nymphs Harris (Hemiptera: Aphididae). *A. pisum* was reared on faba bean *Vicia faba* L. plants under conditions of 22 ± 1 °C, 65 ± 5% RH and 16:8 (L:D) h photoperiod. The seeds of faba bean were purchased online and grown inside cages. *A. pisum* was then introduced to these faba beans to start an aphid laboratory culture. Eggs of *H. axyridis* were removed daily and kept in 9 cm Petri dishes on moistened tissue paper. Hatchlings were transferred to separate Petri dishes to avoid cannibalism. These Petri dishes had folded tissue paper, *A. pisum* nymphs as food and moist cotton as water source. All the insects were kept under controlled laboratory conditions at 70 ± 5% RH, 24 ± 1 °C, and 16:8 (L:D) h photoperiod. All larval, pupal, and adult stages were maintained under same settings as above. The plant materials used were obtained with prior permission, and the present study complies with relevant guidelines and legislation.

The pupae of *S. litura* were purchased online from Henan Jiyuan Baiyun Industrial Co., Ltd, China, in September 2018 and placed in a transparent glass jar (20 cm × 12 cm × 8 cm) and incubated inside the growth chamber (Jiangnan Instruments RXZ-430, Ningbo, China) at settings of 26 ± 1 °C and 70 ± 5% RH under a 16:8 h photoperiod (L: D) to obtain adult populations. Hatchlings from *S. litura* eggs were fed with an artificial diet and cultured until the reach of final instar stage inside a mesh covered transparent circular glass jars (1 L). The artificial diet prepared followed Saljoqi, et al.^37^. The artificial diet (semi-solid) consisted of yeast powder (24 g), ascorbic acid (2.35 g), methyl-4-hydroxy benzoate (1.5 g), distilled water 550 mL, kidney bean flour (150 g), agar (8.4 g), sorbic acid (0.75 g), formaldehyde solution (1 mL) and streptomycin (0.75 g). The final caterpillars were isolated and subsequently cultured until maturity inside similar jars with 20% glucose solution as adult’s diet and clean pieces of paper covering jar’s bottom as oviposition substratum. The cotton wool strips (2 cm wide, 7–12 cm long) were placed in jars as a suitable oviposition substrate in order to obtain new oviposition. Oviposition was checked daily. Hatchlings were reared together in a separate Petri dish. Same age larvae were reared together using the same artificial diet as above to maintain population homogeneity.

### Life table and predation trials

The experimental setup used here was a Petri dish (6 cm), and one leaf disc of cotton (*Gossypium hirsutum* L. cv. Varamin) placed on a 0.5 cm thick layer of 1.5% agar to fix the leaf disk. Paired *H. axyridis* adults were separated from the stock culture and reared with *S. litura* eggs in transparent plastic cups (9 cm in diameter and 7 cm in height) covered with fine nylon netting for ventilation under conditions of 70 ± 5% RH, 24 ± 1 °C, and 16:8 (L:D) h photophase for two consecutive generations to avoid interbreeding. Hundred *H. axyridis* eggs laid within 24 h were collected and transferred to Petri dish for the life table trials. Hatchlings were transferred to new Petri dishes and reared on sufficient eggs until pupation. The duration and viability of the individuals were recorded daily. Instar changes were evaluated based on the exuvia presence. The experiment was performed at four different temperatures (i.e., 15, 20, 25 and 30 °C), 70 ± 5% RH, and 16:8 (Light: Dark) h photoperiod. The temperature regimes tested here represent the thermal conditions that *H. axyridis* encounters in different protected plantations and field crops in tropical and temperate regions^38^. Experimental cohorts were generated as fifty individuals per temperature. Survival rate and time to hatch were recorded for each stage in each temperature treatment. The number of surviving larvae and the total developmental period from larva to pupa were also recorded. Predation was recorded for both larval and adult stages of the predator. After eclosion, the predator larvae were fed with *S. litura* eggs until pupation. In addition to this, male and female adults that emerged from the pupae on the same day from a given temperature were numbered and coupled in the insect boxes. Adult pairs were monitored to ensure that mating occurred, and time allowed for mating event was ~ 10 h a day, and no prey was made available during mating events throughout the trial duration. One or a few matings are reported sufficient for coccinellids females to fertilize all eggs. No eggs provision during mating events was meant to avoid any potential disturbance during this activity. After mating, the pairs were separated and shifted cautiously to their respective Petri dishes with eggs. The life span of an adult male is shorter than female, hence extra pairs of insects were raised in parallel to supply supplementary males for mating depending on the need at each temperature, simultaneously. The number of eggs laid per female including the cannibalized ones and adult mortality was noted twice daily until all female adults died. About 400–500 eggs of *S. litura* were supplied daily to the larvae and adults on cotton leaves. The counted numbers of *S. litura* eggs were fed to each stage of the predator. Predation data were recorded daily as numbers of eggsdamaged or consumed. Petri dishes and boxes were cleaned daily with 75% ethanol.

### Data analysis

The data were analyzed by using TWOSEX-MSChart^39^ according to age-stage, two-sex life table theory^14,40^. The age stage-specific survival rate (*s*_*xj*_), age-specific survival rate (*l*_*x*_), age-specific fecundity (*m*_*x*_), the net maternity (*l*_*x*_*m*_*x*_), age-stage life expectancy (*e*_*xj*_), age-stage reproductive value (*v*_*xj*_), population and reproduction parameters were calculated accordingly. All oviposited eggs per female were counted to calculate the fecundity.

The age-stage survival rate *s*_*xj*_ was calculated as:

1 $$s_{xj} = \frac{{n_{xj} }}{{n_{01} }} \,$$

where *n*_01_ is the number of individuals used at the beginning of the life table study and *n*_*xj*_ is the number of individuals surviving to age *x* and stage *j*^41^.

The age-specific survival rate (*l*_*x*_*)* was calculated as:

2 $$l_{x} = \sum\limits_{j = 1}^{\beta } {s_{xj} } \,$$

and age-specific fecundity (*m*_*x*_) were obtained from the following formula, where *β* is the number of stage *s*_*xj*_ is the age-stage specific survival rate, i.e. the possibility that a new insect (newly hatched individual) will live or exist to age *x* and stage *j*.

3 $$m_{x} = \frac{{\sum\limits_{j = 1}^{\beta } {s_{xj} f_{xj} } }}{{\sum\limits_{j = 1}^{\beta } {s_{xj} } }}$$

The net reproductive rate (*R*_0_) is defined as the total number of offspring that an insect or individual can produce during its lifetime and was calculated as:

4 $$R_{{0}} = \sum\limits_{x = 0}^{\infty } {l_{x} m_{x} }$$

The intrinsic rate of increase (*r*) was then predicted iteratively following the Euler–Lotka equation with age indexed from 0^42^ by the following formula,

5 $$\sum\limits_{x = 0}^{\infty } {e^{{ - r\left( {x + 1} \right)}} l_{x} m_{x} } = 1$$

The finite rate of increase (*λ*) was calculated as:

6 $$\lambda = e^{r}$$

The mean generation time (*T*) is the time duration that a population needs to increase to ${\text{R}}_{{0}}$-fold of its size at the stable age-stage distribution, and was calculated as:

7 $$T = \frac{{\ln R_{0} }}{r}$$

Age-stage life expectancy (*e*_*xj*_) is the duration that an insect of *x* age and *j* stage is predicted to live^43^ as:

8 $$e_{xj} = \sum\limits_{i = x}^{\infty } {\sum\limits_{y = j}^{\beta } {s^{\prime}_{iy} } }$$

where $s^{\prime}_{iy}$ is the probability that an individual of age *x* and stage *j* will survive to age *i* and stage *y* and it is calculated by assuming *s*_*xj*_ = 1.

The age-stage reproductive value (*v*_*xj*_) was defined as the contribution of individuals of age *x* and stage *j* to the future population and is calculated according to Tuan et al.^44,45^.

9 $$v_{xj} = \frac{{e^{{r\left( {x + 1} \right)}} }}{{s_{xj} }}\sum\limits_{i = x}^{\infty } {e^{{ - r\left( {i + 1} \right)}} } \sum\limits_{y = j}^{\beta } {s^{\prime}_{iy} f_{iy} }$$

Raw data on daily predation rates were analyzed according to^46^ by CONSUME-MSChart^47^. The age-specific predation rate (*k*_*x*_, the number of prey consumed by the surviving individuals at age *x*), age-stage specific predation rate (*c*_*xj*_, the mean predation rate of individuals at age *x* and stage *j*), age-specific net predation rate (*q*_*x*_), net predation rate (*C*_0_), transformation rate (*Q*_*p*_), finite predation rate (*ω*) and stable predation rate (*ψ*) were calculated.

The age-stage specific predation rate *c*_*xj*_ was calculated as:

10 $$c_{xj} = \frac{{\sum\limits_{i = 1}^{{n_{xj} }} {d_{xj,i} } }}{{n_{xj} }}$$

where *d*_*xj*,*i*_ is the calculated predation rate for the *i*th individual at age *x* and stage *j*.

The age specific predation rate (*k*_*x*_) was calculated as:

11 $$k_{x} = \frac{{\sum\limits_{j = 1}^{\beta } {s_{xj} c_{xj} } }}{{\sum\limits_{j = 1}^{\beta } {s_{xj} } }}$$

The age specific net predation rate (*q*_*x*_) by considering survival rate was calculated as:

12 $$q_{x} = l_{x} k_{x} = \sum\limits_{j = 1}^{\beta } {s_{xj} c_{xj} }$$

The net predation rate (*C*_0_) is defined as the number of prey consumed by an average individual predator during its lifetime. It includes all individuals that died in the preadult stages and those that survived to the adult stage. It was calculated as:

13 $$C_{0} = \sum\limits_{x = 0}^{\infty } {\sum\limits_{j = 1}^{\beta } {s_{xj} c_{xj} } } = \sum\limits_{x = 0}^{\infty } {l_{x} k_{x} }$$

The transformation rate (*Q*_*p*_) is the number of prey needed for a predator to produce a single offspring. It was calculated as:

14 $$Q_{p} = \frac{{C_{0} }}{{R_{0} }}$$

The finite predation rate (*ω*) was calculated according to Yu, et al.^30^ as:

15 $$\omega = \lambda \psi = \lambda \sum\limits_{x = 0}^{\infty } {\sum\limits_{j = 1}^{\beta } {a_{xj} c_{xj} } }$$

where *a*_*xj*_ is the proportion of individuals belonging to age *x* and stage *j* in a stable age-stage distribution, *ψ* is the stable predation rate.

Variances and standard errors (SE) of all parameters were determined by using 100,000 bootstraps replications^48–50^. We used paired bootstrap tests to compare differences among the temperatures at 5% significance level based on the confidence interval of difference^51^. All graphs were plotted with SigmaPlot (ver. 12, Systat Software, Palo Alto, CA).

### Population and predation projection

The population and predation projection began with 10 pairs of *Harmonia axyridis* at all temperatures and it was projected for 90 d to obtain the total population size assuming a scenario without suppression by biotic and/or abiotic factors and predation potential with unlimited prey, i.e., *S. litura* eggs.

The total population size at time t was calculated as:

16 $$N\left( t \right) = \sum\limits_{j = 1}^{\beta } {\sum\limits_{x = 0}^{\infty } {n_{xj, \, t} } }$$

where *n*_*xj,t*_ is the number of individuals of age *x* and stage *j* at time *t*^52^.

The predation potential at time *t* was calculated following Huang, et al.^52^:

17 $$P\left( t \right) = \sum\limits_{j = 1}^{\beta } {\sum\limits_{x = 0}^{\infty } {c_{xj} n_{xj, \, t} } }$$

To reveal the variability of both projections, we sorted the 100,000 bootstrap results of the finite rate of increase (*λ*) to find the 2.5th and 97.5th percentiles, i.e., the 2,500th and 97,500th sorted bootstrap samples. We then used the bootstrap life table samples that generated the 2.5th and 97.5th percentiles of the finite rate of increase (*λ*) to project the population to represent the confidence interval of the projected populations^52^. Projections were made based on the method described by^41,53^ and via TIMING- MSChart computer program^54^.

## Results

### Development time of each stage of *H. axyridis*

Increasing temperature from 15 to 30 °C significantly (*P* < 0.05) reduced development times for eggs, larvae, pupae and adult stages of the *H. axyridis* (Table 1). When temperature increased from 15 to 30 °C, the developmental time decreased from 7.08 to 2.24 days for first instar, 5.33 to 1.64 days for second instar, 5.19 to 2.11 days for third instar, 8.85 to 3.21 days for fourth instar, 88.32 to 37.61 days for adult males, and 105.94 to 46.65 days for adult females. Increasing temperature from 15 to 30 °C significantly reduced the duration of the total juvenile stages. Male and female longevities were far greater at colder temperature (15 °C) than at warmer temperatures (25–30 °C). The total preadult stage was 15.39 d at 30 °C and 44.26 d at 15 °C.

**Table 1:** Duration of each stage and preadult survival rate (Mean ± SE) of *Harmonia axyridis* on *Spodoptera litura* eggs at four different temperatures.

Variables	Temperature							
	15 °C		20 °C		25 °C		30 °C	
	*n*	Mean ± SE	*n*	Mean ± SE	*n*	Mean ± SE	*n*	Mean ± SE
Egg (day)	50	6.02 ± 0.11 a	50	4.72 ± 0.09 b	50	3.00 ± 0.07 c	49	2.47 ± 0.07 d
First-instar (day)	49	7.08 ± 0.12 a	48	4.50 ± 0.09 b	49	3.23 ± 0.06 c	46	2.24 ± 0.06 d
Second-instar (day)	49	5.33 ± 0.10 a	48	4.00 ± 0.07 b	44	2.62 ± 0.07 c	44	1.64 ± 0.07 d
Third-instar (day)	48	5.19 ± 0.09 a	48	4.67 ± 0.09 b	44	2.98 ± 0.06 c	44	2.11 ± 0.08 d
Fourth-instar (day)	47	8.85 ± 0.20 a	46	7.02 ± 0.14 b	42	3.90 ± 0.09 c	43	3.21 ± 0.06 d
Pupa (day)	46	11.80 ± 0.24 a	46	8.02 ± 0.12 b	41	4.88 ± 0.08 c	38	3.74 ± 0.07 d
Total preadult stage (day)	46	44.26 ± 0.41 a	46	32.91 ± 0.29 b	41	20.63 ± 0.15 c	38	15.39 ± 0.12 d
Female longevity (day)	18	105.94 ± 3.85 a	24	81.25 ± 1.86 b	19	70.58 ± 2.68 c	20	46.65 ± 1.63 d
Male longevity (day)	28	88.32 ± 2.76 a	22	71.68 ± 2.28 b	22	61.82 ± 1.99 c	18	37.61 ± 0.99 d
Female Total longevity (day)	18	150.61 ± 3.96 a	24	113.83 ± 1.89 b	19	91.21 ± 2.76 c	20	62.05 ± 1.62 d
Male Total longevity (day)	28	132.32 ± 2.69 a	22	104.95 ± 2.21 b	22	82.45 ± 1.96 c	18	53.00 ± 1.03 d
Pre-adult survival rate(s_a_)	50	0.92 ± 0.04 a	50	0.92 ± 0.04 a	50	0.82 ± 0.05 ab	50	0.76 ± 0.06 b

Standard errors were estimated by using the bootstrap technique with 100,000 resampling. Difference was compared using the paired bootstrap test (*P* < 0.05). The means within a row followed by a different letter indicate significant differences among the temperatures.

The *s*_*xj*_ of *H. axyridis* fed with *S. litura* eggs at four constant temperatures is presented in Supplementary Fig. S1. Results showed an increase in temperature reduced the survival rates of new hatchlings. The survival was of 161 and 171 days at 15 °C, 124 and 131 d at 20 °C, 95 and 112 d at 25 °C, and 61 and 74 d at 30 °C (Supplementary Fig. S1d), with survival peaks of 0.56 and 0.36 at 15 °C, 0.44 and 0.48 at 20 °C, 0.44 and 0.38 at 25 °C, and 0.36 and 0.40, at 30 °C for male and female adults, respectively.

### Reproduction and population parameters of *H. axyridis*

The temperature effects were assessed on the estimated intrinsic rate of increase (r), finite rate of increase (*λ*), net reproductive rate (*R*_0_), and mean generation time (T) for *H. axyridis* feeding on *S. litura* eggs by applying the bootstrap technique with 100,000 resamplings (Table 2). Population parameters i.e. *r*, *λ*, *R*_*0,*_ and *T* of *H. axyridis* on *S. litura* eggs under influence of temperature are presented in Table 2. The *r* (the intrinsic rate of increase) and *λ* (finite rate of increase) highest values were 0.1378 d^−1^ and 1.1477 d^−1^, respectively, at 30 °C. The shortest mean generation time (35.55 d) was obtained at 30 °C. The lowest *r* and *λ* values (0.0662 d^−1^ and 1.0685 d^−1^) and the longest generation time (79.84 d) were observed at 15 °C. The net reproductive rate, *R*_0_, was significantly higher (237.96) at 20 °C when compared with other temperatures.

**Table 2:** Reproduction and population parameters (Mean ± SE) of *Harmonia axyridis* fed on *Spodoptera litura* eggs at four different temperatures.

Parameters^a^	Temperature			
	15 °C	20 °C	25 °C	30 °C
	Mean ± SE	Mean ± SE	Mean ± SE	Mean ± SE
*R*_0_ (offspring/individual)	198.02 ± 37.71 ab	237.96 ± 35.64 a	184.20 ± 34.12 ab	133.92 ± 23.96 b
*r* (day^−1^)	0.0662 ± 0.0032 d	0.0843 ± 0.0029 c	0.1067 ± 0.0046 b	0.1378 ± 0.0059 a
*λ* (day^−1^)	1.0685 ± 0.0034 d	1.0880 ± 0.0031 c	1.1126 ± 0.0052 b	1.1477 ± 0.0067 a
*T* (day)	79.84 ± 1.87 a	64.90 ± 0.71 b	48.89 ± 0.74 c	35.55 ± 0.54 d
*F* (eggs/female)	550.06 ± 15.89 a	495.75 ± 14.25 b	484.74 ± 19.13 b	334.80 ± 13.89 c
O_*d*_ (day)	26.94 ± 0.93 a	22.83 ± 0.63 b	19.32 ± 0.77 c	16.65 ± 0.73 d
APOP (day)	11.50 ± 0.76 ab	12.88 ± 0.60 a	10.00 ± 0.28 b	8.85 ± 0.26 c
TPOP (day)	56.17 ± 1.11 a	45.46 ± 0.68 b	30.63 ± 0.35 c	24.25 ± 0.34 d

Standard errors were estimated by using the bootstrap technique with 100,000 resampling. Difference was compared using the paired bootstrap test (*P* < 0.05). The means within a row followed by a different lowercase letters indicate significant differences among the temperatures.

*APOP* adult preoviposition period, *TPOP* total preoviposition period.

^a^*R*_0_ = net reproductive rate; *r* = intrinsic rate of increase; *λ* = finite rate of increase; *T* = mean generation time; *F* = fecundity; O_*d*_ = oviposition days.

The mean fecundity and oviposition days decreased with warming temperatures. Fecundity was highest at 15 °C with 550.06 eggs per female, which decreased as the temperature increased i.e., 495.75 eggs at 20 °C, 484.74 eggs at 25 °C, and 334.80 eggs at 30 °C. The oviposition days were significantly shortest at 30 °C with 16.65 d and longest at 15 °C with 26.94 d. Temperature impacts were obvious on the adult pre-oviposition period (APOP) and total pre-oviposition period (TPOP) (Table 2). APOP of females lasted shortest at 30 °C (i.e., 8.85 days) and longest at 20 °C (i.e., 12.88 d). TPOP was shortest at 30 °C (i.e., 24.25 d) and colder temperatures prolonged it from 56.17 d at 15 °C to 45.46 d at 20 °C, and 30.63 d at 25 °C.

**Figure 1:**
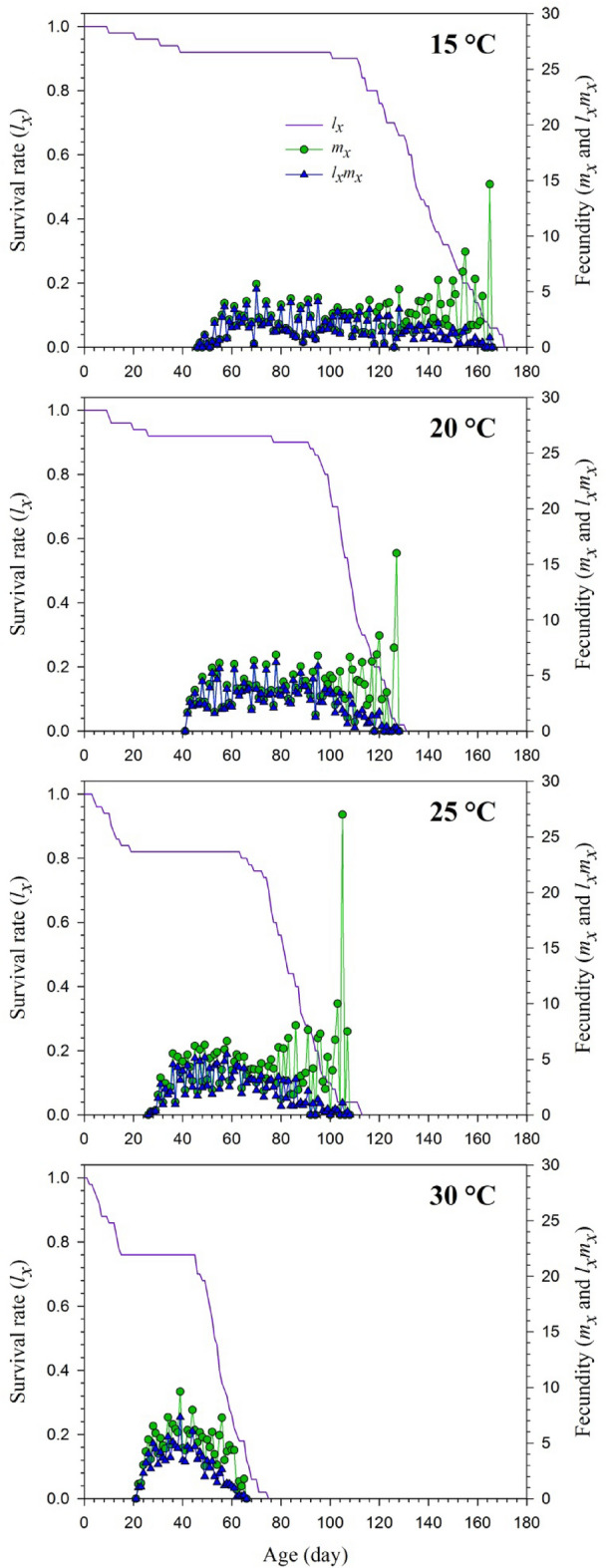
Age-specific survival rate (*l*_*x*_), age-specific fecundity (*m*_*x*_), and age-specific maternity (*l*_*x*_*m*_*x*_) of the *Harmonia axyridis* feeding on *Spodoptera litura* eggs at different temperatures.

Figure 1 shows *l*_*x*_ (age-specific survival rate), *m*_*x*_ (total fecundity of population), and *l*_*x*_*m*_*x*_ (net maternity) of *H. axyridis* population fed with *S. litura* eggs. The effects of temperature were observed on all the mentioned parameters. The *l*_*x*_ is described as the simple form of the *s*_*xj*_ curves. The age-specific survival rate of *H. axyridis* population fed with *S. litura eggs* at 15 °C decreased to 0.92 on the 47th d when the first fecundity occurred. At 20 °C, the first fecundity was obtained on the 42nd d with 0.92 survival rates. The fecundity of the population was seen early at 25 °C on the 27th d with 0.82 lx and 22nd d with survival rate decreasing to 0.76 at 30 °C. The *l*_*x*_*m*_*x*_ curves depended on *l*_*x*_ and *m*_*x*_ values. The highest *l*_*x*_*m*_*x*_ peaks in *H. axyridis* populations were 5.24 (on 70th d), 6.18 (on 78th d), 5.44 (on 58th d) and 7.32 (on 39th) (Fig. 1).

The e_*xj*_ (age-stage life expectancy) of *H. axyridis* fed with *S. litura* eggs at four constant temperatures are plotted in Supplementary Fig. S2. The e_*xj*_ estimates the length of duration of the newly hatched individual. Results showed temperature impacts e_*xj*_ curve of *H. axyridis*, but with varied degrees, i.e. highest life expectancy values for females and males were 111.61 d and 93.32 d at 15 °C, 84.83 d and 75.96 d at 20 °C, 72.21 d and 63.46 d at 25 °C, 72.21 d and 63.46 d at 30 °C (Supplementary Fig. S2). Supplementary Fig. S3 presents v_*xj*_ (age-stage-specific reproduction) of *H. axyridis* fed with *S. litura* eggs at four constant temperatures. The *v*_*xj*_ tells the contribution every individual will likely have in the next progeny. The curves show that temperature change also changes the age stage-specific reproduction. The highest v_*xj*_ value of *H. axyridis* female fed with *S. litura* eggs was 102.16 eggs/d (on 61st d) at 15 °C which changed to 95.53 eggs/day (on 61st d) at 20 °C, 92.54 eggs/d (on 42nd d) at 25 °C and 85.11eggs/d (on 34th d) at 30 °C (Supplementary Fig. S3).

### Predation rate of *H. axyridis*

Daily predation of 1st-4th instar increased at all tested temperatures, exponentially. Daily mean prey consumption during the pre-adult stages was significantly different between temperatures as 22.57, 39.87, 44.86 and 52.46 eggs/larva at 15, 20, 25 and 30 °C, respectively (Table 3). Female predators consumed significantly more than did males within the same temperature and total daily predation of adults varied significantly with 79.58, 130.50, 172.02 and 198.67 eggs/adults at 15, 20, 25 and 30 °C, respectively (Table 3). The age-stage specific predation rate (*c*_*xj*_) of *H. axyridis* which means the mean predation rate of individuals at age *x* and stage *j* was presented in Supplementary Fig. S4. While the long survival durations increase total predations in lower temperatures i.e. 15 and 20 °C, daily predation through the stages was generally higher at 25 and 30 °C. The net predation rate (*C*_0_) was greatest i.e., 10,466.28 and 10,139.38 at 20 and 25 °C than those at 15 and 30 °C with 7935.54 and 7126.36 eggs/individual, respectively (Table 4). There was no significant difference in transformation rate (*Q*_*p*_) at all tested temperatures. The transformation rate (*Q*_*p*_) was highest at 25 °C, which indicates that *H. axyridis* requires 55.05 *S. litura* eggs for the production of each egg at 20 °C. Stable and finite predation rates differed among all tested temperatures. The highest values for both parameters were obtained at 30 °C, whereas lowest values were at 15 °C.

**Table 3:** Stage daily predation (*D*_*j*_) (preys/individual) (Mean ± SE) of *Harmonia axyridis* fed on *Spodoptera litura* eggs at four different temperatures.

Stages	Temperature							
	15 °C		20 °C		25 °C		30 °C	
	*n*	Mean ± SE	*n*	Mean ± SE	*n*	Mean ± SE	*n*	Mean ± SE
First-instar	50	8.02 ± 0.27 d	50	13.62 ± 0.39 c	50	17.43 ± 0.45 b	49	22.94 ± 0.58 a
Second-instar	49	17.56 ± 0.31 d	48	20.58 ± 0.35 c	48	30.43 ± 0.83 b	46	36.61 ± 1.15 a
Third-instar	49	32.65 ± 0.68 d	48	40.44 ± 1.04 c	47	56.20 ± 1.97 b	44	70.29 ± 0.99 a
Fourth-instar	48	77.08 ± 1.77 c	48	140.95 ± 2.98 b	44	166.04 ± 3.35 a	44	175.83 ± 4.58 a
Preadult	50	22.57 ± 0.41 d	50	39.87 ± 0.74 c	50	44.86 ± 1.08 b	50	52.46 ± 1.38 a
Adult (Female)	18	86.30 ± 1.56 dA	24	146.79 ± 2.31 cA	19	193.91 ± 2.48 bA	20	223.41 ± 4.08 aA
Adult (Male)	28	74.40 ± 0.71 dB	22	110.36 ± 2.73 cB	22	150.43 ± 2.74 bB	18	164.58 ± 1.32 aB
Adults	46	79.58 ± 1.18 d	46	130.50 ± 3.23 c	41	172.02 ± 3.94 b	38	198.67 ± 5.32 a

Standard errors were estimated by using the bootstrap technique with 100,000 resampling. Difference was compared using the paired bootstrap test (*P* < 0.05). The means within a row followed by a different lowercase letters indicate significant differences among the temperatures, while different uppercase letters within the same column indicate significant differences between female and male adult stage daily predation.

**Table 4:** Predation rates (Mean ± SE) of *Harmonia axyridis* fed on *Spodoptera litura* eggs at four different temperatures.

Predation rate parameters	Temperature			
	15 °C	20 °C	25 °C	30 °C
	Mean ± SE	Mean ± SE	Mean ± SE	Mean ± SE
Net Predation Rate, *C*_0_ (preys/individual)	7935.54 ± 402.31 b	10,466.28 ± 544.76 a	10,139.38 ± 753.86 a	7126.36 ± 626.73 b
Transformation Rate, *Q*_*p*_ (*C*_0_/*R*_0_)	40.0744 ± 7.6199 a	43.9834 ± 5.7392 a	55.0455 ± 9.0397 a	53.2136 ± 7.2403 a
Finite Predation Rate, *ω* (preys)	19.1684 ± 0.4193 d	34.3995 ± 0.8513 c	50.2287 ± 1.7168 b	61.9303 ± 2.4656 a
Stable Predation Rate, *ψ* (preys)	17.9399 ± 0.3735 d	31.6179 ± 0.7482 c	45.1458 ± 1.4551 b	53.9603 ± 1.9577 a

Standard errors were estimated by using the bootstrap technique with 100,000 resampling. Difference was compared using the paired bootstrap test (*P* < 0.05). The means within a row followed by a different lowercase letters indicate significant differences among the temperatures.

**Figure 2:**
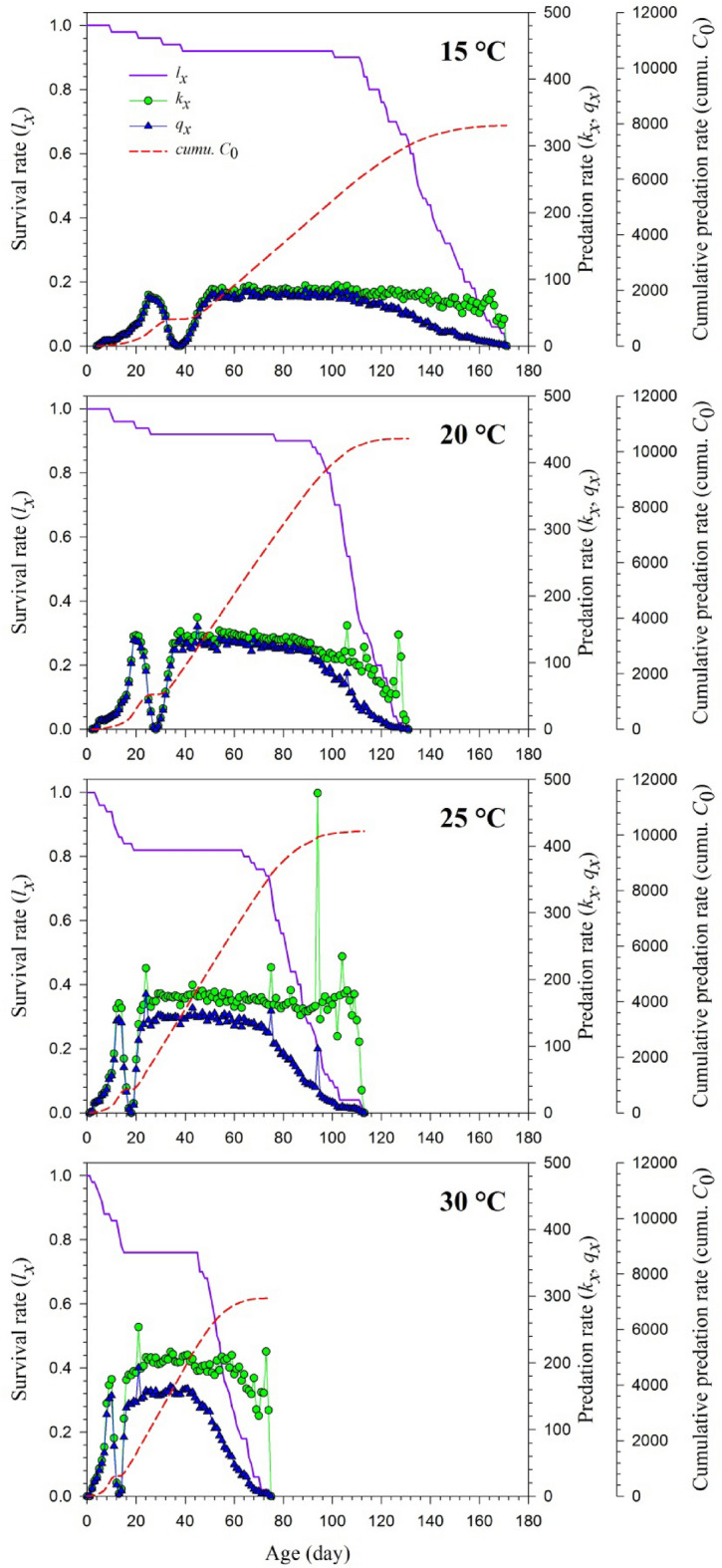
Age-specific survival rate (*l*_*x*_), age-specific predation rate (*k*_*x*_), age-specific net predation rate (*q*_*x*_) cumulative net predation rate (*C*_0_) of *Harmonia axyridis* feeding on *Spodoptera litura* eggs at different temperatures.

Age-specific predation rate (*k*_*x*_), age-specific net predation rate (*q*_*x*_) cumulative net predation rate (*C*_*0*_) for *H. axyridis* feeding on *S. litura* eggs at four different temperatures were presented in Fig. 2. The *k*_*x*_ gives the average number of *S. litura* eggs eaten by *H*. *axyridis* of age *x*, while *q*_*x*_ gives the weighted number of aphids eaten by *H*. *axyridis* of age *x*. As *H*. *axyridis* feed with *S. litura* eggs only in the larval and adult period, the increase in *k*_*x*_ and *q*_*x*_ was linked to survival rates until the adult period with a stepwise reduction beginning from the adult period. There were two gaps in the curves for *k*_*x*_ and *q*_*x*_ at all tested temperatures due to non-predator i.e., egg and pupa stages of the *H. axyridis.* Cumulative predation rates were 7935 (on 171st d), 10,466 (on 131st d), 10,139 (on 113rd d) and 7126 (on 75th d) at 15, 20, 25 and 30 °C, respectively (Fig. 2).

### Population and predation projection

**Figure 3:**
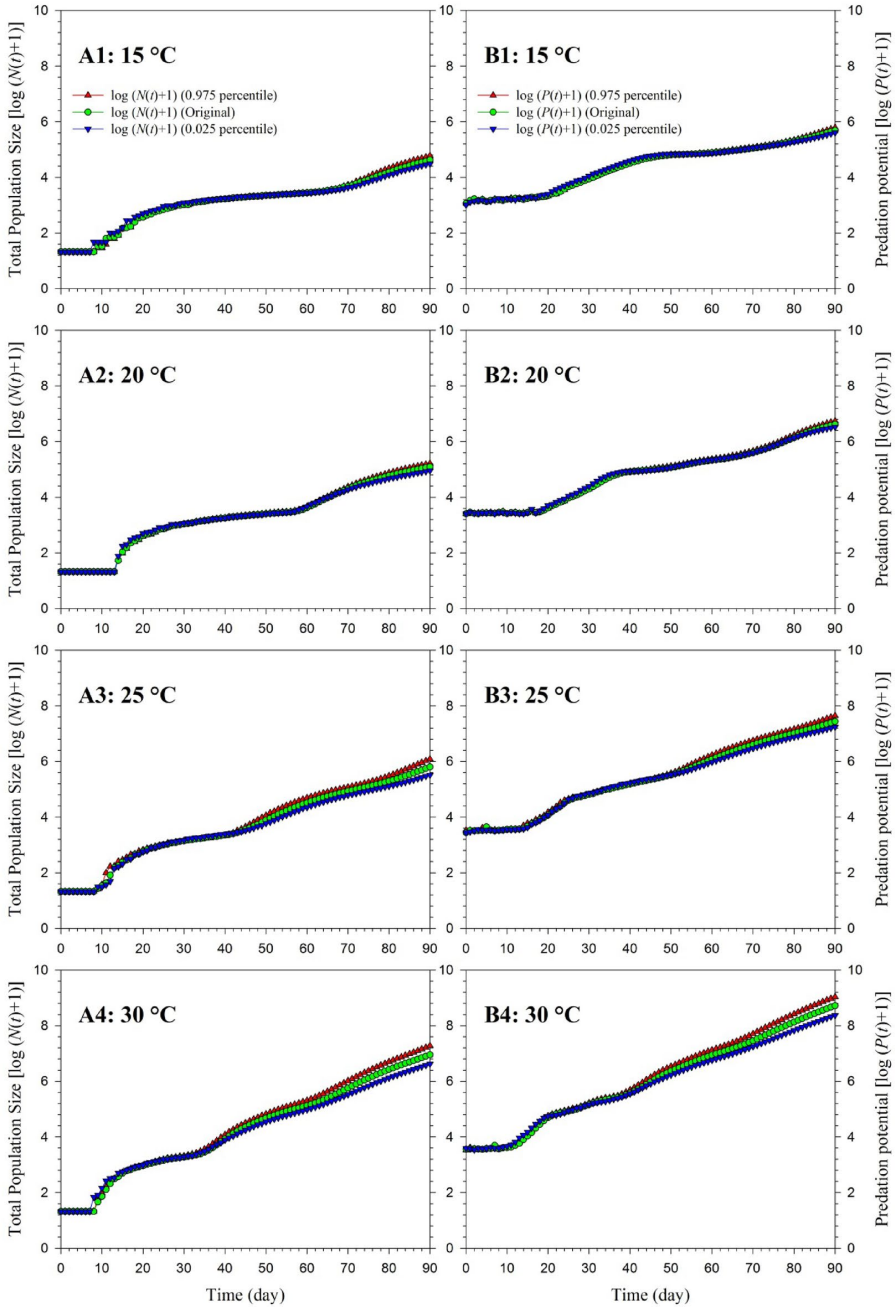
Population and predation projection over a 90-day period of *Harmonia axyridis* feeding on *Spodoptera litura* eggs at different temperatures (the original cohort and the cohorts constructed based on the 2.5 and 97.5% percentiles of *λ,* finite rate of increase are all in log base 10).

Both the population and predation projection for 90 days began with 10 *H. axyridis* pairs at all temperatures (Fig. 3). The variability of both population growth and predation potential was assessed by utilizing the life tables that generated the 0.025th and 0.975th percentiles of *λ* of 100,000 bootstrap results. The total population size of *H. axyridis* was highest at 30 °C, projected to exceed 8.9 × 10^6^ individuals, whereas the population at 15 °C was estimated at 4.3 × 10^4^ individuals. The total population at 25 and 30 °C increased significantly than those at 15 and 20 °C. At lower temperatures (i.e., 15 and 20 °C), the curves of different stages tended to be straight lines (Fig. 3). Total consumption of *S. litura* eggs was highest and projected to reach 5.24 × 10^8^, while egg consumption was approximately 4.9 × 10^5^ at 15 °C after 90 days (Fig. 3).

## Discussion

Coccinellid species are generalist predators and the type of prey they consume shows great impacts on their fitness and predation. The amount and quality of food is very important because it influences directly biological aspects of predators^55^. When ingested food is scarce or inferior quality, the development time usually increases and the reproductive rate, i.e., oviposition, fecundity and fertility decreases^56^. Given the relevance of coccinellids for biological control, much attention has been paid to documenting feeding habits within this group. Food that provides better development and reproduction is considered essential for coccinellids. Many aphids, psyllids and mealybugs are important preys. Many other energy sources as preys that prolong survival are characterized as alternative^57^. Coccinellidae are shown to reproduce even more on non-aphid preys in some studies, which demonstrates the importance of alternative resource^58,59^. Eggs of lepidopterans were the best diet for *H. axridis* because shorter duration and higher survival of the larval stage of this predator was evident on the eggs^34,60^. Abdel‐Salam and Abdel‐Baky^34^ estimated the *R*_0_ of *H.* *axyridis* when feeding on frozen and fresh eggs of the *Sitotrega cerealella*, and showed moth eggs to be a better diet based on findings obtained by applying the female age-specific life table and adult age. This shows that scales and chorion of eggs were not barriers for larval as well as adults feeding, as reported for *H.* *axyridis* with *S. litura* eggs^35^. The current study is the first reporting on the survivorship, development, reproduction, and longevity of *H. axyridis* on *S. litura* eggs at different temperatures. The current study reveals evidence of temperature influence on the development and predation of *H. axyridis*. We also used computer projections based on bootstrap percentile confidence intervals to better understand the variability of population growth and predation parameters.

In our findings, the development and reproduction of *H. axyridis* on *S. litura* eggs were temperature-dependent. The total larval duration was the longest at 15 °C, intermediate at 20 and 25 °C, and the shortest at 30 °C. The development periods of the egg, preadult stages, as well as total development period of the adult stages (Females: Males), were significantly different in length with the four different temperatures tested. The shortest developmental periods for the preadult and adult stages of *H. axyridis* occurred at 25 °C and 30 °C. The shorter development duration of preadult and adult *H. axyridis* at higher temperatures (i.e., 30 °C) is most likely due to increased attack and metabolic rate in response to increased food consumption and energy requirements, allowing the predator to rapidly advance to the next stage. The mean longevities of male and female adults also prolonged at 15 °C and reduced with warming temperatures, in accordance with De Oliveira Ramos, et al.^60^, who under similar thermal conditions reared *H. axyridis* on different prey, *Anagasta kuehniella* (Zeller) (Lepidoptera: Pyralidae). de Castro-Guedes, et al.^33^ reported that female *H. axyridis* could survive on average 91.13 ± 25.53 d on the eggs of *A. kuehniella* at 25 °C. Abdel‐Salam and Abdel‐Baky^34^ reported female *H. axyridis* to survive on average 62.2 ± 2.70 d on *S. cerealella* eggs at 24 °C. The fecundity of female *H. axyridis* fed on different lepidopterans eggs also changed with temperature. For example, the fecundity of *H. axyridis* increased from 806.00 ± 78.4 to 1.685.2 ± 209.6 per female as the temperature increased from 18 to 30 °C. Similarly, the fecundity was recorded 715.3 ± 33.62 per female, when *H. axyridis* was allowed to feed on *S. cerealella* eggs at 24 °C. Accelerated metabolic rate, which leads to increased food consumption to satisfy energy requirements may has allowed the predator complete its development rapidly and advance to the next stage, relatively in a short period of time under influence of warmer temperatures.

Insect population dynamics rely heavily on fecundity^61,^ was the highest at 15 °C (i.e., 550.06 eggs) in our findings, suggesting *S. litura* to be better for the development of *H. axyridis* at lower temperature than higher temperatures. The findings support *H. axyridis* can lay more eggs at optimum temperature, which is in consistence with the previous research^27^. Castro, et al.^27^ reported that *H. axyridis* fed with *C. atlantica* showed the greatest fecundity at 15 °C (i.e., 805.7 ± 127.3) than when fed on the same prey at 20 and 25 °C (i.e., 608.5 ± 113.7 and 614 ± 129.2, respectively). In our experiment, the fecundity comparatively reduced at higher temperatures but not the predation that increased with warmer temperatures. Higher temperatures can reduce reproductive fitness of *H. axyridis*, and other researchers have reported similar findings^62^, which means *H. axyridis* survives thermally complex and diverse environments with such a trade off. This research was conducted using total fecundity without the exclusion of unhatched eggs, however it was showed by Mou, et al.^63^ that the life table and predation parameters differ depending on whether the egg viability factor is included or excluded. As coccinellid's eggs are not always fertile, and as that fertility rate varies with temperature and predator diet, with resulting consequences on biological procedures^27^, other investigations should look at fertility as well as fecundity to gain a better understanding of this predator's biological performance. The duration of adult pre-oviposition days including APOP and TPOP was shortened with higher temperatures. These results are in agreement with those of other researchers such as Castro, et al.^27^, who reported the highest and the lowest APOP and TPOP for *H. axyridis* when it was fed with *C. atlantica* at 15 °C (6.10 and 82.40 d) and 25 °C (5.80 and 76.87 d), respectively.

We found that predation of *H. axyridis* was also temperature-dependent and increased with warmer temperature. Warming is expected to expedite predation because it boosts the predator's capacity to grab and handle prey in response to increased metabolic rate that can result from increased development and fostered growth^12^. In comparison to first three instars, the fourth larval instar and female *H. axyridis* consumed more *S. litura* eggs and showed the maximum predation capacity at these stages. Other researchers have reported that the predation rate of preadult stages of *H. axyridis* generally increased with larval sizes^12,35^. Females consumed more eggs than did males because females need to oviposit. The sexes differed in our findings in a similar way. The female age-specific fecundity correlates with the trend of predation rate. Similar results have also been reported by another researcher who showed fourth instar of *Hippodamia variegata* (Coleoptera: Coccinellidae) to consume significantly more *Aphis fabae* Scopoli (Hemiptera: Aphididae) than other stages of the predator at 23 °C^64^.

This study concludes that *H. axyridis *development depends on the temperature and predation varies with predator stage, temperature, and resource being consumed. On the eggs of *S. litura*, longevity , reproductive rate and mean generation time of *H. axyridis *were much improved at 15 and 25 °C than at 25 and 30 °C. Lower temperatures can thus be favorable for mass rearing the predator. Warmer temperatures (30 °C) can lead to an accelerated development and growth of this predator, which means an increased predation rate. These findings may help with devising effective biocontrol programs against *S. litura* using *H. axyridis* as the predator, probably by identifying potential predator stage and optimal release time. This study further provides useful information about the future consequences of warming on this predator development and predation parameters, which can help attune bio-based IPM programs according to changing climates^11,12.^ Hance, et al.^65^ reported that divergences between thermal optimums of predators and preys may promote disturbance of geographical or temporal synchronization, increasing the risk of pest outbreaks. Temperature limits pest distribution; however, warming has resulted in more suitable disciplines for pests, e.g., insect range expansions. Our experiments used a thermal maximum of 30 °C, and temperatures in Asia can reach as high as > 40 °C for short periods of time at midday during hot summer^66^. Presumably, *S. litura* in this region is locally adapted to short bouts of high heat and can withstand it, although additional research is needed to confirm that host eggs exposed to high heat are suitable for predator development. Although the present study was performed under laboratory conditions, it may still provide information that can complement field studies. Future field experiments under controlled conditions are recommended to provide a much realistic view of the effectiveness of *H. axyridis* in the field against this serious pest.

## Supplementary Information

Supplementary Information.

## Data Availability

The datasets used and/or analysed during the current study available from the corresponding author on reasonable request.

## References

[1] Ahmad M, Saleem MA, Sayyed AH (2009). Efficacy of insecticide mixtures against pyrethroid-and organophosphate-resistant populations of *Spodoptera litura* (Lepidoptera: Noctuidae). Pest. Manage. Sci..

[2] Shekhawat SS, Shafiq AM, Basri M (2018). Effect of host plants on life table parameters of *Spodoptera litura*. Ind. J. Pure Appl. Biosci..

[3] Sang S (2016). Cross-resistance and baseline susceptibility of *Spodoptera litura* (Fabricius) (Lepidoptera: Noctuidae) to cyantraniliprole in the south of China. Pest Manag. Sci..

[4] Ortega DS, Bacca T, Silva APN, Canal NA, Haddi K (2021). Control failure and insecticides resistance in populations of *Rhyzopertha dominica* (Coleoptera: Bostrichidae) from Colombia. J. Stored Prod. Res..

[5] Li L (2022). Pest biological control: Goals throughout my life. Annu. Rev. Entomol..

[6] Razaq, M., Shah, F. M., Ahmad, S. & Afzal, M. in Pest management for agronomic crops. *Agronomic Crops* (ed. Hasanuzzaman M.) 365–384 (Springer, 2019).

[7] Shah, F. M. & Razaq, M. in From agriculture to sustainable agriculture: Prospects for improving pest management in industrial revolution 4.0. *Handbook of Smart Materials, Technologies, and Devices: Applications of Industry 4.0. Cham.* (ed. Cham) 1–18 (Springer, 2020).

[8] Razaq, M. & Shah, F. M. in Biopesticides for management of arthropod pests and weeds. *Biopesticides. Biopesticides Voulme 2: Advances in Bioinoculants* 7–18 (Elsevier, 2022).

[9] Kishinevsky M, Keasar T, Bar-Massada A (2017). Parasitoid abundance on plants: Effects of host abundance, plant species, and plant flowering state. Arthropod-Plant Interact..

[10] Islam Y (2022). Age-stage, two-sex life table and predation parameters of *Harmonia axyridis* Pallas (Coleoptera: Coccinellidae), reared on *Acyrthosiphon pisum* (Harris) (Hemiptera: Aphididae), at four different temperatures. Crop Prot..

[11] Furlong MJ, Zalucki MP (2017). Climate change and biological control: The consequences of increasing temperatures on host–parasitoid interactions. Curr. Opin. Insect Sci..

[12] Islam Y (2021). Functional response of *Harmonia axyridis* preying on *Acyrthosiphon pisum* nymphs: The effect of temperature. Sci. Rep..

[13] Keva O (2021). Increasing temperature and productivity change biomass, trophic pyramids and community-level omega-3 fatty acid content in subarctic lake food webs. Glo. Change Bio..

[14] Chi H (2020). Age-stage, two-sex life table: An introduction to theory, data analysis, and application. Entomol. Gen..

[15] Guedes C (2013). Preferência alimentar e estratégias de alimentação em Coccinellidae (Coleoptera). Oecol. Aust..

[16] Hodek, I. & Honêk, A. *Ecology of coccinellidae*. Vol. 54 464 (Kulver Academic Publisher, 2013).

[17] Sutherland AM, Parrella MP (2009). Mycophagy in *Coccinellidae*: Review and synthesis. Biol. Control.

[18] Hagen K, Ks H (1974). The significance of predaceous *Coccinellidae* in biological and integrated control of insects. Entomophaga.

[19] Jawad, D. S., Rashid, Y. D. & Hamzah, A. G. in *IOP Conference Series: Earth and Environmental Science.* 012029 (IOP Publishing).

[20] Kumari S, Suroshe SS, Kumar D, Budhlakoti N, Yana V (2021). Foraging behaviour of *Scymnus coccivora* Ayyar against cotton mealybug *Phenacoccus solenopsis* Tinsley. Saudi J. Biol. Sci..

[21] Alloush AA (2019). Developmental duration and predation rate of the coccidophagous coccinellid *Rhyzobius lophanthae* (Blaisdell) (Coleoptera: Coccinellidae) on *Aspidiotus nerii* Bouche. Bull. Entomol. Res..

[22] Koch R, Hutchison W, Venette R, Heimpel G (2003). Susceptibility of immature monarch butterfly, *Danaus plexippus* (Lepidoptera: Nymphalidae: Danainae), to predation by *Harmonia axyridis* (Coleoptera: Coccinellidae). Biol. Control.

[23] Islam Y, Shah FM, Güncan A, DeLong JP, Zhou X (2022). Functional response of *Harmonia axyridis* to the larvae of *Spodoptera litura*: The combined effect of temperatures and prey instars. Front. Plant Sci..

[24] Dixon, A. F. G. & Dixon, A. E. *Insect predator-prey dynamics: ladybird beetles and biological control*. (Cambridge University Press, 2000).

[25] Thompson S (1999). Nutrition and culture of entomophagous insects. Annu. Rev. Entomol..

[26] Chaudhary DD, Kumar B, Mishra G (2022). Functional response in *Coccinellid* beetles (Coleoptera: Coccinellidae) is modified by prey-density experience. Can. Entomol..

[27] Castro C, Almeida L, Penteado S (2011). The impact of temperature on biological aspects and life table of *Harmonia axyridis* (Pallas) (Coleoptera: Coccinellidae). Fla. Entomol..

[28] Noman QM, Shah FM, Mahmood K, Razaq M (2021). Population dynamics of Tephritid fruit flies in citrus and mango orchards of Multan, Southern Punjab, Pakistan. Pakistan J. Zool..

[29] Eliopoulos P, Stathas G (2008). Life tables of *Habrobracon hebetor* (Hymenoptera: Braconidae) parasitizing *Anagasta kuehniella* and *Plodia interpunctella* (Lepidoptera: Pyralidae): Effect of host density. J. Econ. Entomol..

[30] Yu J-Z, Chi H, Chen B-H (2013). Comparison of the life tables and predation rates of *Harmonia dimidiata* (F.) (Coleoptera: Coccinellidae) fed on *Aphis gossypii* Glover (Hemiptera: Aphididae) at different temperatures. Biol. Control.

[31] Roy HE, Ten Brown PM (2015). years of invasion: *Harmonia axyridis* (Pallas)(Coleoptera: Coccinellidae) in Britain. Ecol. Entomol..

[32] Koch R (2003). The multicolored Asian lady beetle, *Harmonia axyridis*: a review of its biology, uses in biological control, and non-target impacts. J. Insect. Sci..

[33] de Castro-Guedes CF, de Almeida LM, do Rocio CPS, Moura MO (2016). Effect of different diets on biology, reproductive variables and life and fertility tables of *Harmonia axyridis* (Pallas) (Coleoptera, Coccinellidae). Rev. Bras. Entomol..

[34] Abdel-Salam A, Abdel-Baky N (2001). Life table and biological studies of *Harmonia axyridis* Pallas (Col., Coccinellidae) reared on the grain moth eggs of *Sitotroga cerealella* Olivier (Lep., Gelechiidae). J. Appl. Entomol..

[35] Islam Y (2020). Temperature-dependent functional response of *Harmonia axyridis* (Coleoptera: Coccinellidae) on the eggs of *Spodoptera litura* (Lepidoptera: Noctuidae) in laboratory. Insects.

[36] Di N (2021). Predatory ability of *Harmonia axyridis* (Coleoptera: Coccinellidae) and *Orius sauteri* (Hemiptera: Anthocoridae) for suppression of fall armyworm *Spodoptera frugiperda* (Lepidoptera: Noctuidae). Insects.

[37] Saljoqi A-U-R, Khan J, Ali G (2015). Rearing of *Spodoptera litura* (Fabricius) on different artificial diets and its parasitization with *Trichogramma chilonis* (Ishii). Pak. J. Zool..

[38] Brown PM (2011). The global spread of *Harmonia axyridis* (Coleoptera: Coccinellidae): Distribution, dispersal and routes of invasion. Biocontrol.

[39] Chi, H. TWOSEX-MSChart: a computer program for the age-stage, two-sex life table analysis. Available from http://140.120.197.173/ecology/Download/TWOSEX-MSChart-B100000.rar. (2022).

[40] Chi H (1988). Life-table analysis incorporating both sexes and variable development rates among individuals. Environ. Entomol..

[41] Chi H, Liu H (1985). Two new methods for the study of insect population ecology. Bull. Inst. Zool. Acad. Sin..

[42] Goodman D (1982). Optimal life histories, optimal notation, and the value of reproductive value. Am. Nat..

[43] Chi H, Su H-Y (2006). Age-stage, two-sex life tables of *Aphidius gifuensis* (Ashmead) (Hymenoptera: Braconidae) and its host *Myzus persicae* (Sulzer) (Homoptera: Aphididae) with mathematical proof of the relationship between female fecundity and the net reproductive rate. Environ. Entomol..

[44] Tuan SJ, Lee CC, Chi H (2014). Population and damage projection of *Spodoptera litura* (F.) on peanuts (*Arachis hypogaea* L.) under different conditions using the age-stage, two-sex life table. Pest Manag. Sci..

[45] Tuan SJ, Lee CC, Chi H (2014). Erratum: Population and damage projection of *Spodoptera litura* (F.) on peanuts (*Arachis hypogaea* L.) under different conditions using the age-stage, two-sex life table. Pest Manag. Sci..

[46] Chi H, Yang T-C (2003). Two-sex life table and predation rate of *Propylaea japonica* Thunberg (Coleoptera: Coccinellidae) fed on *Myzus persicae* (Sulzer)(Homoptera: Aphididae). Environ. Entomol..

[47] Chi, H. CONSUME-MSChart: a computer program for consumption rate analysis based on the age stage, two-sex life table analysis. http://140.120.197.173/ecology/Download/CONSUME-MSChart.rar. (2022).

[48] Akca I, Ayvaz T, Yazici E, Smith CL, Chi H (2015). Demography and population projection of *Aphis fabae* (Hemiptera: Aphididae): With additional comments on life table research criteria. J. Econ. Entomol..

[49] Polat Akköprü E, Atlıhan R, Okut H, Chi H (2015). Demographic assessment of plant cultivar resistance to insect pests: A case study of the dusky-veined walnut aphid (Hemiptera: Callaphididae) on five walnut cultivars. J. Econ. Entomol..

[50] Huang YB, Chi H (2012). Age-stage, two-sex life tables of *Bactrocera cucurbitae* (Coquillett) (Diptera: Tephritidae) with a discussion on the problem of applying female age-specific life tables to insect populations. Insect Sci..

[51] Wei M (2020). Demography of *Cacopsylla chinensis* (Hemiptera: Psyllidae) reared on four cultivars of *Pyrus bretschneideri* (Rosales: Rosaceae) and *P. communis* pears with estimations of confidence intervals of specific life table statistics. J. Econ. Entomol..

[52] Huang H-W, Chi H, Smith CL (2018). Linking demography and consumption of *Henosepilachna vigintioctopunctata* (Coleoptera: Coccinellidae) fed on *Solanum photeinocarpum* (Solanales: Solanaceae): with a new method to project the uncertainty of population growth and consumption. J. Econ. Entomol..

[53] Chi H (1990). Timing of control based on the stage structure of pest populations: a simulation approach. J. Econ. Entomol..

[54] Chi, H. TIMING-MSChart: a computer program for the population projection based on age-stage, two-sex life table. (http://140.120.197.173/Ecology/Download/TIMING-MSChart.rar). (2022).

[55] Mignault MP, Roy M, Brodeur J (2006). Soybean aphid predators in Quebec and the suitability of *Aphis glycines* as prey for three Coccinellidae. BioControl.

[56] Brown M (2003). Intraguild responses of aphid predators on apple to the invasion of an exotic species, *Harmonia axyridis*. BioControl.

[57] Pervez A, Chandra S, Kumar R (2021). Effect of dietary history on intraguild predation and cannibalism of ladybirds’ eggs. Int. J. Trop. Insect Sci..

[58] Lundgren JG (2009). Nutritional aspects of non-prey foods in the life histories of predaceous *Coccinellidae*. Biol. Control.

[59] Yu JZ, Chen BH, Güncan A, Atlıhan R, Gökçe A, Smith CL, Gümü E, Chi H (2018). Demography and mass-rearing *Harmonia dimidiata *(Coleoptera: Coccinellidae) using *Aphis gossypii* (Hemiptera: Aphididae) and eggs of *Bactrocera dorsalis* (Diptera: Tephritidae). J. Econ. Entomol..

[60] De Oliveira RT, dos Santos-Cividanes TM, Cividanes FJ, da Conceic L (2014). *Harmonia axyridis* Pallas (Coleoptera: Coccinellidae): Biological aspects and thermal requirements. Adv. Entomol..

[61] Ali S (2020). Using a two-sex life table tool to calculate the fitness of *Orius strigicollis* as a predator of *Pectinophora gossypiella*. Insects.

[62] Merene Y (2015). Population dynamics and damages of onion thrips (Thripstabaci)(Thysanoptera: Thripidae) on onion in Northeastern Ethiopia. J. Entomol. Nematol..

[63] Mou DF, Lee CC, Smith C, Chi H (2015). Using viable eggs to accurately determine the demographic and predation potential of *Harmonia dimidiata* (Coleoptera: Coccinellidae). J. Appl. Entomol..

[64] Farhadi R, Allahyari H, Chi H (2011). Life table and predation capacity of *Hippodamia variegata* (Coleoptera: Coccinellidae) feeding on *Aphis fabae* (Hemiptera: Aphididae). Biol. Control.

[65] Hance T, van Baaren J, Vernon P, Boivin G (2007). Impact of extreme temperatures on parasitoids in a climate change perspective. Annu. Rev. Entomol..

[66] Ma X, Zhu J, Yan W, Zhao C (2021). Projections of desertification trends in Central Asia under global warming scenarios. Sci. Total Environ..

