# Supplementary material for: Effect of temperature on the life cycle of Harmonia axyridis (Pallas), and its predation rate on the Spodoptera litura (Fabricius) eggs
Source: Sci Rep. 2022 Sep 12;12:15303. doi: 10.1038/s41598-022-18166-z (PMC9468180; doi:10.1038/s41598-022-18166-z)
Supplement: Supplementary file 1 — Supplementary Information. [file 41598_2022_18166_MOESM1_ESM.docx]

**Supplementary Figures Legends**

**Life table analysis and predation of *Harmonia axyridis* (Pallas), feeding on the eggs of *Spodoptera litura* (Fabricius) under four constant temperatures**

Yasir Islam^1^, Ali Güncan^2,^ Xingmiao Zhou^1,^* Afifa Naeem^3^, Farhan Mahmood Shah^4,†*^,

^1^Hubei Insect Resources Utilization and Sustainable Pest Management Key Laboratory, College of Plant Science and Technology, Huazhong Agricultural University, Wuhan 430070, China

^2^Department of Plant Protection, Faculty of Agriculture, Ordu University, 52200 Ordu, Turkey (ORCID: 0000-0003-1765-648X, e-mail: [guncan.ali@gmail.com](mailto:guncan.ali@gmail.com))

^3^Entomological Research Institute, Ayub Agricultural Research Institute, Faisalabad, Punjab, Pakistan

^4^Department of Entomology, Faculty of Agricultural Sciences and Technology, Bahauddin Zakariya University, Multan 60000, Pakistan (ORCID: 0000-0002-6123-1860)

^†^ New Address: National Center for Natural Products Research, The University of Mississippi, University, MS, USA

*Correspondence to: FMS: [farhanshah0009@yahoo.com](mailto:farhanshah0009@yahoo.com); XZ: x[mzhou@mail.hzau.edu.cn](mailto:mzhou@mail.hzau.edu.cn)

**
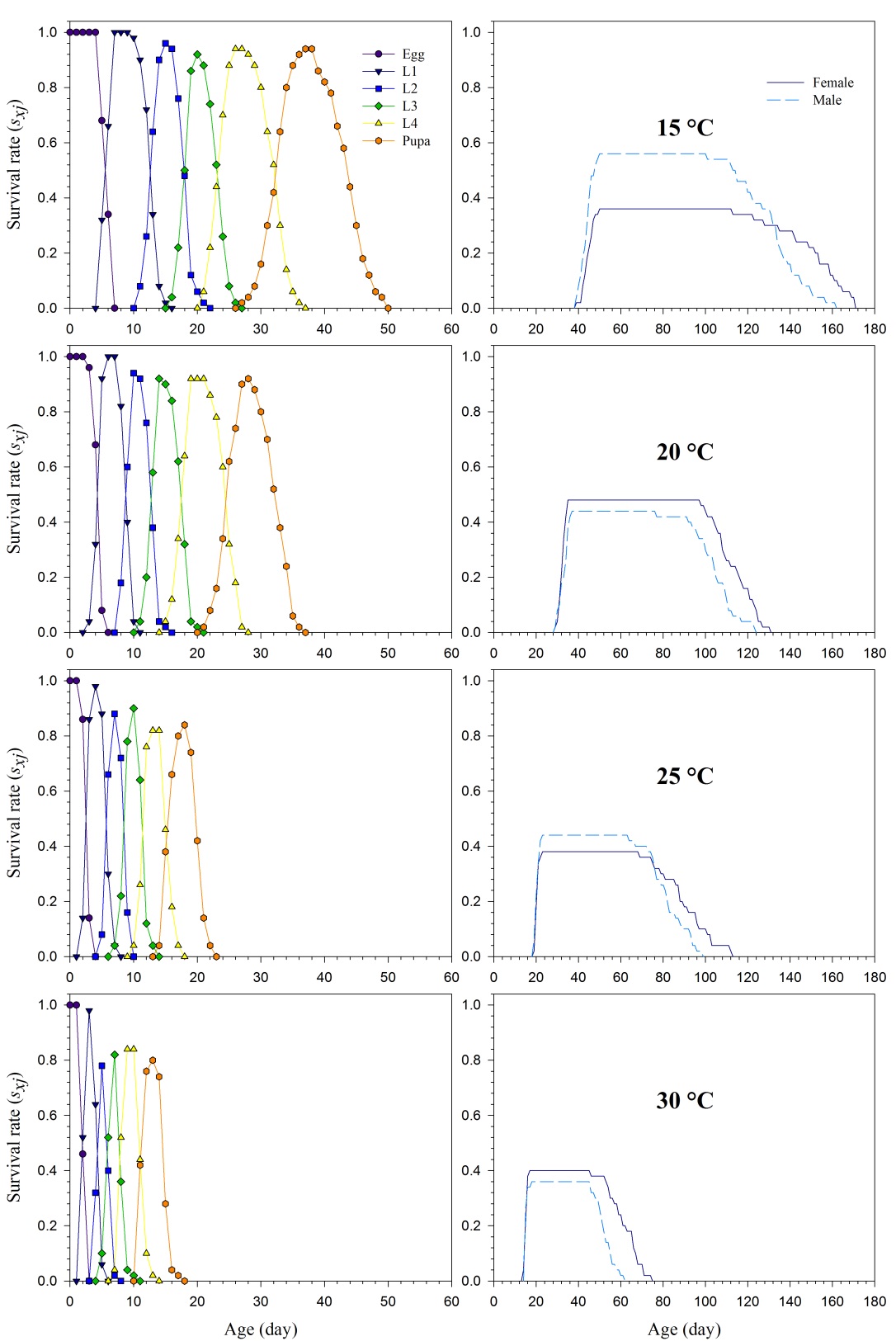
**

Figure S1 Age-stage specific survival rate (*s_x_*) of the *Harmonia axyridis* feeding on *Spodoptera litura* eggs at different temperatures)

**
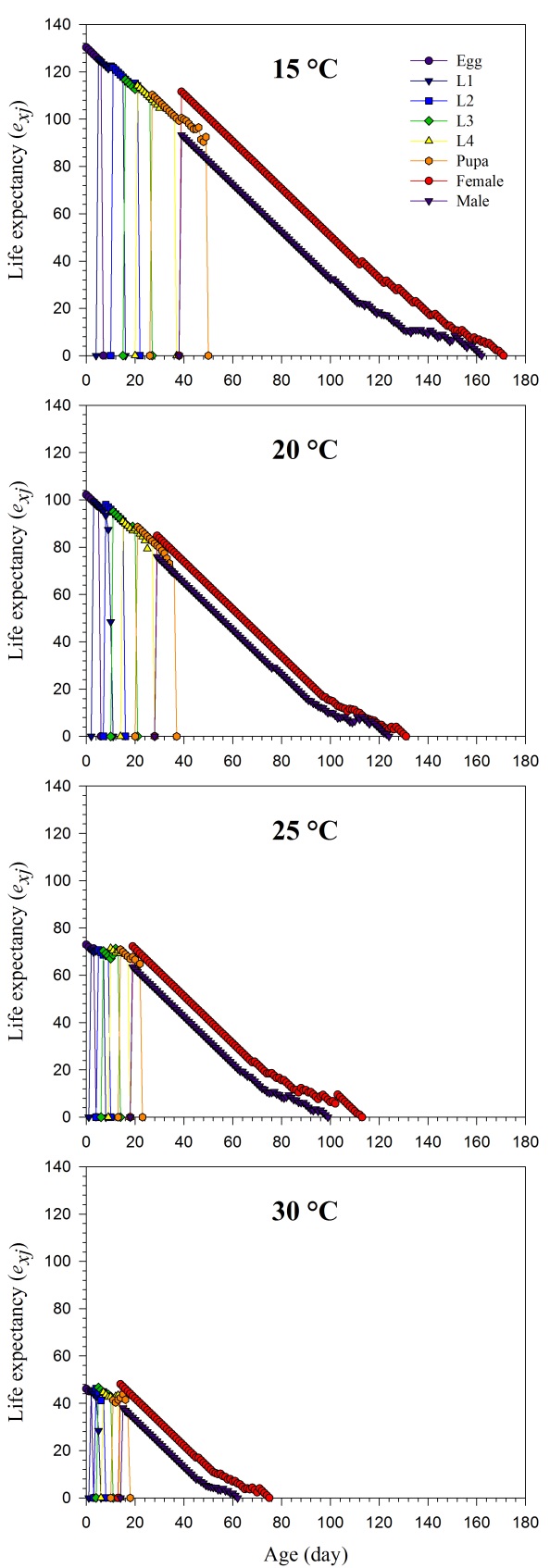
**

Figure S2 Age-stage life expectancy (e*_xj_*) of the *Harmonia axyridis* feeding on *Spodoptera litura* eggs at different temperatures

**
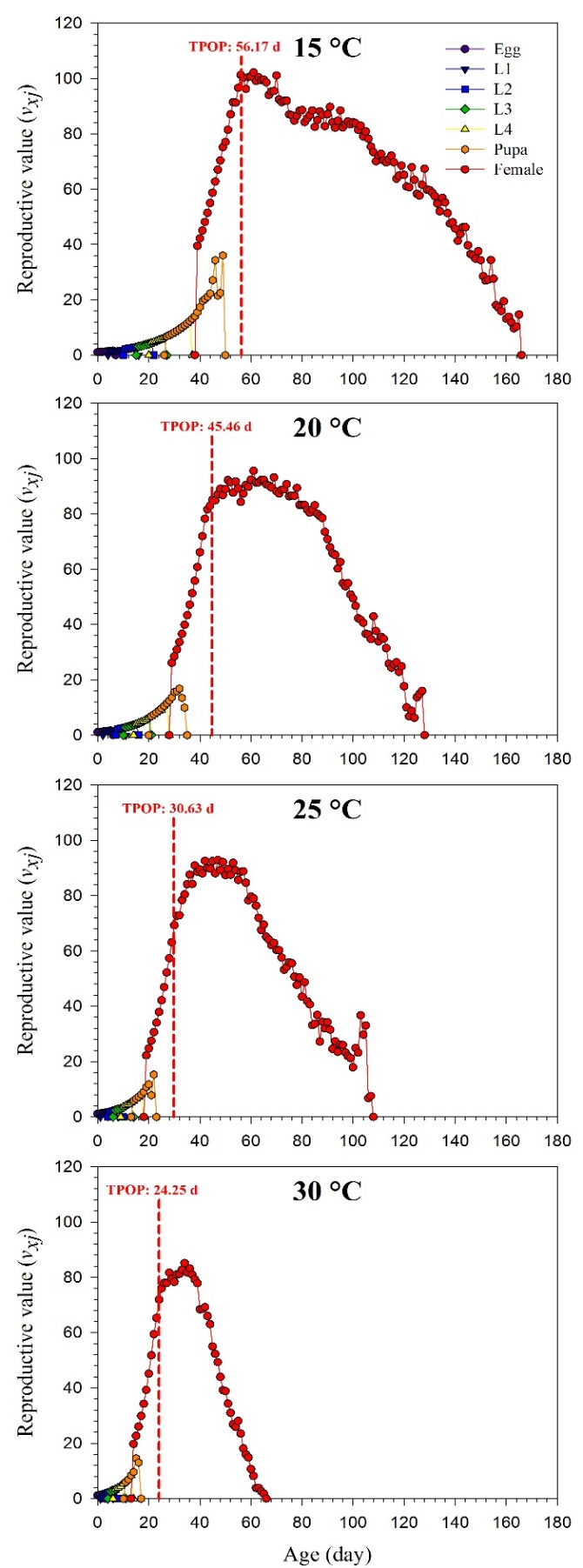
**

Figure S3 Age-stage-specific reproduction (v*_xj_*) of the *Harmonia axyridis* feeding on *Spodoptera litura* eggs at different temperatures (**TPOP**: total preoviposition period)


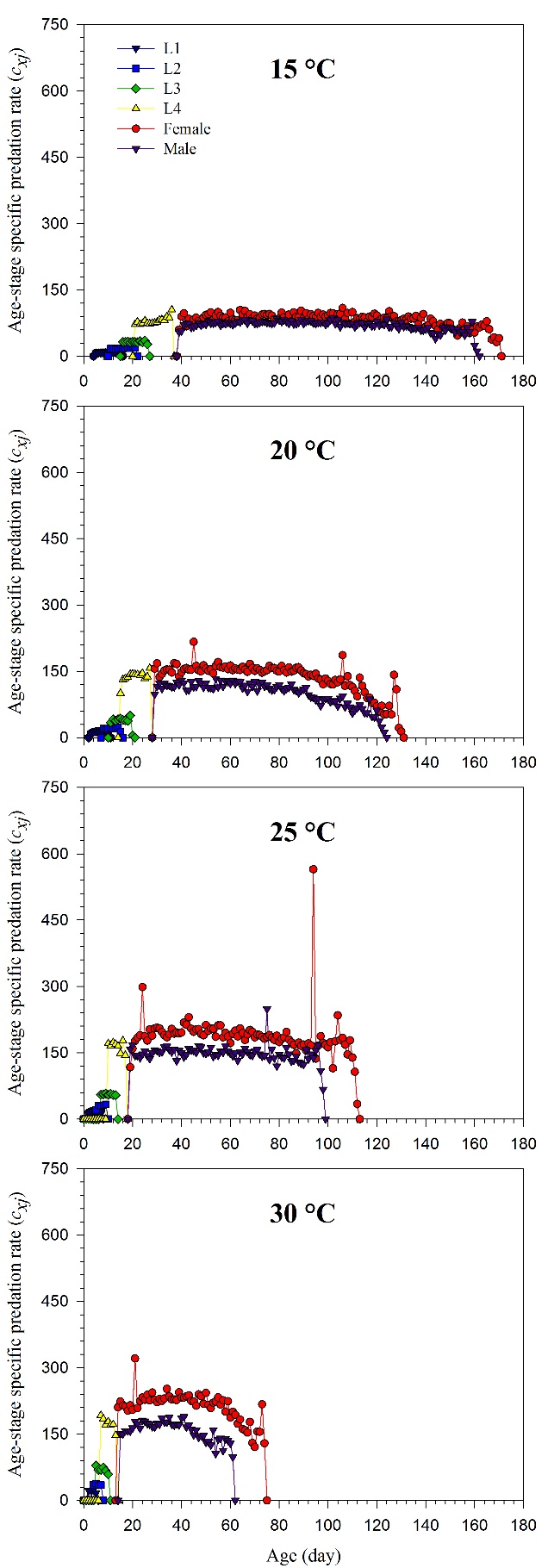


Figure S4 Age-stage specific predation rate (*c_xj_*) of the *Harmonia axyridis* feeding on *Spodoptera litura* eggs at different temperatures
